# Association of Maternal Sexually Transmitted Infections With Risk of Preterm Birth in the United States

**DOI:** 10.1001/jamanetworkopen.2021.33413

**Published:** 2021-11-29

**Authors:** Rui Gao, Buyun Liu, Wenhan Yang, Yuxiao Wu, Bo Wang, Mark K. Santillan, Kelli Ryckman, Donna A. Santillan, Wei Bao

**Affiliations:** 1 Shenzhen Birth Cohort Study Center, Nanshan Maternity and Child Healthcare Hospital of Shenzhen, Shenzhen, China; 2 Department of Epidemiology, University of Iowa College of Public Health, Iowa City; 3 Department of Maternal and Child Health, Sun Yat-sen University School of Public Health, Guangzhou, Guangdong Province, China; 4 Department of Pediatrics, Guangdong Women and Children Hospital, Guangzhou, Guangdong Province, China; 5 Department of Obstetrics & Gynecology, University of Iowa, Iowa City; 6 Center for Hypertension Research, University of Iowa, Iowa City

## Abstract

**Importance:**

Maternal infection has been implicated in the pathogenesis of preterm birth through intrauterine inflammatory response. Chlamydia, gonorrhea, and syphilis are among the most common sexually transmitted infections worldwide, but studies on their association with preterm birth are sparse.

**Objective:**

To examine the association between maternal chlamydia, gonorrhea, and syphilis infections in pregnancy and the risk of preterm birth in a large population-based study in the US.

**Design, Setting, and Participants:**

This population-based retrospective cohort study examined nationwide birth certificate data from the US National Vital Statistics System between 2016 and 2019. All mothers who had a singleton live birth and available data on chlamydia, gonorrhea, or syphilis infection before or during pregnancy and gestational age at birth were included in analysis.

**Exposures:**

Sexually transmitted infection (chlamydia, gonorrhea, or syphilis) occurring before or during pregnancy.

**Main Outcomes and Measures:**

Preterm birth, defined as gestational age less than 37 weeks.

**Results:**

This study included 14 373 023 mothers (mean [SD] age 29 [5.8] years; Hispanic, 3 435 333 [23.9%]; non-Hispanic Asian, 912 425 [6.3%]; non-Hispanic Black, 2 058 006 [14.3%]; and non-Hispanic White, 7 386 568 [51.4%]). Among the mothers, 267 260 (1.9%) had chlamydia, 43 147 (0.3%) had gonorrhea, and 16 321 (0.1%) had syphilis. Among the newborns, 1 146 800 (8.0%) were preterm births. The rate of preterm birth was 9.9%, 12.2%, and 13.3% among women with chlamydia, gonorrhea, and syphilis infection, respectively. After adjustment for sociodemographic and medical and/or health factors, the adjusted odds ratio of preterm birth was 1.03 (95% CI, 1.02-1.04) for chlamydia, 1.11 (95% CI, 1.08-1.15) for gonorrhea, 1.17 (95% CI, 1.11-1.22) for syphilis, and 1.06 (95% CI, 1.05-1.07) for any of these sexually transmitted infections comparing mothers with these conditions and those without.

**Conclusions and Relevance:**

Maternal sexually transmitted infections (gonorrhea, syphilis, or chlamydia) were associated with an increased risk of preterm birth. Pregnant women with sexually transmitted infections before or during pregnancy might benefit from targeted prevention for preterm birth.

## Key Points

**Question:**

Are maternal sexually transmitted infections (gonorrhea, syphilis, or chlamydia) associated with preterm birth?

**Findings:**

In this population-based cohort study using US nationwide birth certificate data and including more than 14 million mother-infant pairs with singleton live births, maternal sexually transmitted infections were associated with an increased risk of preterm birth, especially moderately and very preterm birth.

**Meaning:**

These results suggest that maternal sexually transmitted infections may potentially affect neonatal outcomes.

## Teaser

This cohort study examines US nationwide birth certificate data for associations between 4 common sexually transmitted infections and preterm birth.

## Introduction

Preterm birth, defined as birth occurring before the completion of 37 weeks of gestation,^1,2^ is the leading cause of neonatal morbidity and mortality worldwide.^3^ In the US, preterm birth affects approximately 10% of live-born deliveries, and recent data from the National Vital Statistics System (NVSS) showed an increase in preterm birth prevalence from 2016 to 2019.^4^ Continued efforts are needed to identify modifiable and preventable risk factors of preterm birth.

Maternal infection has been implicated in the pathogenesis of preterm birth through intrauterine inflammatory response.^5,6^ Chlamydia, gonorrhea, and syphilis are among the most common sexually transmitted infections (STIs) worldwide. In parallel with the upward trends in preterm birth, the surveillance reports from the US Centers for Disease Control and Prevention (CDC) showed that the prevalence of chlamydia, gonorrhea, and syphilis have been increasing nationally in the general population from 2013 through 2018 in the US.^7^

Maternal STIs are known to have adverse effects on the developing fetus.^8^ However, studies on the association between maternal STIs and preterm birth are sparse, and the results have been inconclusive.^9,10,11^ Inference from some of those previous studies is challenging because they may not have sufficient statistical power because of the relatively low incidence of maternal STIs. The inconsistent findings from previous studies might also be affected by the differences between the study populations. Studies with data from a large, diverse population and available information on potential confounding factors such as detailed sociodemographic information, prenatal factors, and medical and/or health factors are critical to clarifying how maternal STIs may factor into preterm birth.

In the US, chlamydia, gonorrhea, and syphilis are routinely screened during pregnancy and documented in birth certificates. Therefore, in this retrospective cohort study, we took advantage of nationwide birth certificate data to examine the association of maternal chlamydia, gonorrhea, and syphilis infections with the risk of preterm birth in more than 14 million mother-infant pairs.

## Methods

### Study Population and Data Sources

Federal and state laws require birth certificates to be completed for all births in the US. The NVSS, conducted by the National Center for Health Statistics (NCHS) at the CDC, collects and publishes nationwide data on births from birth certificates according to federal law. Data on live births in all 50 US states and the District of Columbia were collected via the Mother’s Worksheet and the Facility Worksheet.^12,13^ In the Mother’s Worksheet, maternal characteristics are obtained by hospital staff from the mother during birth registration. For the Facility Worksheet, data are obtained directly from the medical records and include medical and health information.^14,15^

In this population-based study, we used birth data from January 1, 2016, to December 31, 2019, including all 14 373 023 mothers who had a live singleton birth and available data on maternal STIs and gestational age at birth. We used data for these years because the 2003 revised version of the standard birth certificate for live births, which contains more information about sociodemographic and health information, was fully implemented nationwide in 2016 and thereafter. This study was approved as exempt from review and informed consent requirements by the institutional review board at the University of Iowa because it used deidentified data. The conduct and reporting of this study followed the Strengthening the Reporting of Observational Studies in Epidemiology (STROBE) reporting guideline for cohort studies.

### Exposure Measurement and Outcome Ascertainment

Information on maternal STIs (ie, gonorrhea, syphilis, and chlamydia) was collected directly from the medical record by a birth information specialist or clinician responsible for completing the medical and health information for the certificate of live birth using the facility worksheet. According to the NVSS Facility Worksheets Guidebook, a maternal infection was documented if it was present “at the time of the pregnancy diagnosis or a confirmed diagnosis during the pregnancy with or without documentation of treatment.”^15^ The prenatal records, labor and delivery nursing admission triage form, medical history and physical examination records, and delivery record during the current pregnancy were checked when the birth information specialist or clinician completed the NVSS facility worksheets.

For the ascertainment of preterm birth, gestational age was calculated using the obstetric estimate of gestation at delivery, which was defined as the best obstetric estimate of the infant’s gestation in completed weeks based on the birth attendant’s final estimate of gestation.^16^ Preterm birth was defined as gestational age less than 37 weeks. We further subdivided the outcome based on gestational age: extremely preterm birth (<28 weeks of gestation), very preterm birth (28-31 weeks of gestation), and moderately preterm birth (32-36 weeks of gestation).^2^

### Covariate Assessment

Information on age, race and ethnicity, education, marital status, height, prepregnancy weight, and smoking during pregnancy were collected using the standardized Mother’s Worksheet during birth registration. Information on parity, previous history of preterm birth, prepregnancy diabetes, prepregnancy hypertension, time of initiation of prenatal care, gestational diabetes, gestational hypertension or preeclampsia, eclampsia, insurance type, and infant sex status was taken from the facility worksheet, which is obtained directly from the medical records.

### Statistical Analysis

We assessed the distribution of sociodemographic and maternal characteristics according to STIs and preterm birth, respectively. Comparisons between categorical variables were tested using χ^2^ tests. We compared the risk of preterm birth for mothers with a specific STI (ie, gonorrhea, syphilis, or chlamydia) with those without this specific STI. We also calculated the risk of preterm birth for mothers with any of the 3 included STIs compared with mothers no STIs.

Multivariable logistic regression models were used to estimate the odds ratios (ORs) and 95% CIs of preterm birth. Multinomial logistic regression models were used to calculate ORs and 95% CIs for the subtypes of preterm birth (ie, extremely preterm birth, very preterm birth, and moderately preterm birth). In model 1, we adjusted for maternal age (<25 years, 25-34 years, ≥35 years), and race and ethnicity (Hispanic, non-Hispanic Asian, non-Hispanic Black, non-Hispanic White, other [including non-Hispanic American Indian or Alaskan Native, non-Hispanic Native Hawaiian or Other Pacific Islander, non-Hispanic more than 1 race, and origin unknown or not stated]). In model 2, we further adjusted for maternal education level (less than high school, high school, more than high school), marital status (yes, no), parity (1 child, 2 children, 3 children, 4 or more children), previous history of preterm birth (yes, no, nulliparous), prepregnancy body mass index (calculated as weight in kilograms divided by height in meters squared) (underweight, <18.5; normal weight, 18.5-24.9; overweight, 25.0-29.9; obesity, ≥30.0), prepregnancy diabetes (yes, no), prepregnancy hypertension (yes, no), smoking status during pregnancy (yes, no), timing of initiation of prenatal care (during first to third month, fourth to sixth month, seventh month or later, no prenatal care), insurance type (Medicaid, private insurance, self-pay, other), and infant sex (male, female). These variables were selected based on univariate analysis, biological plausibility and review of the literature. *R^2^* for model 2 was calculated on the basis of multivariable logistic regression to assess the model fit assessment.

We conducted stratified analyses by age, race, and ethnicity to explore whether the association between STIs and preterm birth differed by these demographic factors. *P* for interaction was calculated on the basis of multivariable logistic regression models with multiplicative interaction terms (age or race and ethnicity × specific STI or any STIs). We conducted sensitivity analyses by excluding pregnant women who did not receive prenatal care or did not have insurance or medical assistance because these women may not have been screened for maternal STIs during pregnancy. In addition, because a woman may become pregnant more than 1 time between 2016 and 2019, we performed a sensitivity analysis using data from each year. We also conducted another sensitivity analysis, which additionally adjusted for pregnancy complications based on model 2. Pregnancy complications were defined as having gestational diabetes, gestational hypertension, preeclampsia, or eclampsia.

Two-sided *P* < .0125 (ie, *P* < .05 divided by 4 based on Bonferroni correction) were considered statistically significant. All statistical analyses were conducted using survey modules of SAS software version 9.4 (SAS Institute).

## Results

This study included 14 373 023 mothers who delivered singleton live births. Their mean (SD) age was 29 (5.8) years old; 3 435 333 mothers [23.9%] identified as Hispanic, 912 425 [6.4%] as non-Hispanic Asian, 2 058 006 [14.3%] as non-Hispanic Black, and 7 386 568 [51.4%] as non-Hispanic White (Table 1). Among the mothers, 267 260 (1.9%) had chlamydia, 43 147 (0.3%) had gonorrhea, and 16 321 (0.1%) had syphilis. Among the newborns, 1 146 800 (8.0%) were categorized as preterm births. The rate of preterm birth was 9.9% (26 393 of 267 260 births), 12.2% (5257 of 43 147), and 13.3% (2169 of 16 321) for women with chlamydia, gonorrhea, and syphilis, respectively. Higher rates of gonorrhea, syphilis, and chlamydia infections were found among women younger than age 25 years (eg, chlamydia infections: 166 216 mothers [4.6%] vs ages 25-34 years, 90 231 [1.1%]), non-Hispanic Black women (87 893 [4.3%] vs non-Hispanic White, 84 546 [1.1%]), women with lower education (less than high school, 71 425 [3.8%] vs more than high school, 81 060 [0.9%]), women who were unmarried (213 506 [4.1%] vs married, 44 160 [0.6%]), women who smoked during pregnancy (37 271 [3.9%] vs no, 227 557 [1.7%]), or women who needed Medicaid (197 806 [3.3%] vs private insurance, 49 084 [0.7%]) (Table 1). Population characteristics according to preterm birth and preterm birth subcategories are depicted in eTable 1 in the Supplement.

**Table 1.** Characteristics of the Study Population According to the Presence of Maternal Sexually Transmitted Infections

Variables	Maternal infections, No. (%)^a^						
	Total, No. (%) (N = 14 373 023)	Chlamydia		Gonorrhea		Syphilis	
		No (n = 14 105 763)	Yes (n = 267 260)	No (n = 14 329 876)	Yes (n = 43 147)	No (n = 14 356 702)	Yes (n = 16 321)
Age, y							
<25	3 575 607 (24.9)	3 409 391 (24.2)	166 216 (62.2)	3 550 819 (24.8)	24 788 (57.5)	3 570 133 (24.9)	5474 (33.5)
25-34	8 270 420 (57.5)	8 180 189 (58.0)	90 231 (33.8)	8 254 220 (57.5)	16 200 (37.6)	8 261 972 (57.6)	8448 (51.8)
≥35	2 526 996 (17.6)	2 516 183 (17.8)	10 813 (4.1)	2 524 837 (17.6)	2159 (29.9)	2 524 597 (17.6)	2399 (14.7)
Race or ethnicity							
Hispanic	3 435 333 (23.9)	3 362 417 (23.8)	72 916 (27.3)	3 428 363 (23.9)	6970 (16.2)	3 430 996 (23.9)	4337 (26.6)
Non-Hispanic							
Asian	912 425 (6.4)	908 483 (6.4)	3942 (1.5)	912 103 (6.4)	322 (0.8)	912 042 (6.4)	383 (2.4)
Black	2 058 006 (14.3)	1 970 113 (14.0)	87 893 (32.9)	2 037 514 (14.2)	20 492 (47.5)	2 051 174 (14.3)	6832 (41.9)
White	7 386 568 (51.4)	7 302 022 (51.8)	84 546 (31.6)	7 374 540 (51.5)	12 028 (27.9)	7 382 831 (51.4)	3737 (22.9)
Other^b^	580 691 (4.0)	562 728 (4.0)	17 963 (6.7)	577 356 (4.0)	3335 (7.7)	579 659 (4.0)	1032 (6.3)
Education levels							
Lower than high school	1 864 949 (13.0)	1 793 524 (12.7)	71 425 (26.7)	1 852 021 (12.9)	12 928 (30.0)	1 860 136 (13.0)	4813 (29.5)
High school	3 655 819 (25.4)	3 543 601 (25.1)	112 218 (42.0)	3 637 451 (25.4)	18 368 (42.6)	3 649 694 (25.4)	6125 (37.5)
Higher than high school	8 667 305 (60.3)	8 586 245 (60.9)	81 060 (30.3)	8 655 904 (60.4)	11 401 (26.4)	8 662 239 (60.3)	5066 (31.0)
Missing	184 950 (1.3)	182 393 (1.3)	2557 (1.0)	184 500 (1.3)	450 (1.0)	184 633 (1.3)	317 (1.9)
Marital status							
Yes	7 797 727 (54.3)	7 753 567 (55.0)	44 160 (16.5)	7 792 563 (54.4)	5164 (12.0)	7 794 123 (54.3)	3604 (22.1)
No	5 256 032 (36.6)	5 042 526 (35.8)	213 506 (79.9)	5 219 646 (36.4)	36 386 (84.3)	5 245 064 (36.5)	10 968 (67.2)
Missing	1 319 264 (9.2)	1 309 670 (9.3)	9594 (3.6)	1 317 667 (9.2)	1597 (3.7)	1 317 515 (9.2)	1749 (10.7)
Parity							
1	5 522 842 (38.4)	5 396 802 (38.3)	126 040 (47.2)	5 506 025 (38.4)	16 817 (39.0)	5 517 850 (38.4)	4992 (30.6)
2	4 582 467 (31.9)	4 510 668 (32.0)	71 799 (26.9)	4 570 720 (31.9)	11 747 (27.2)	4 578 223 (31.9)	4244 (26.0)
3	2 437 738 (17.0)	2 399 556 (17.0)	38 182 (14.3)	2 430 336 (17.0)	7402 (17.2)	2 434 544 (17.0)	3194 (19.6)
≥4	1 793 349 (12.5)	1 762 615 (12.5)	30 734 (11.5)	1 786 264 (12.5)	7085 (16.4)	1 789 506 (12.5)	3843 (23.6)
Missing	36 627 (0.3)	36 122 (0.3)	505 (0.2)	36 531 (0.3)	96 (0.2)	36 579 (0.3)	48 (0.3)
Previous preterm birth							
Yes	472 138 (3.3)	460 057 (3.3)	12 081 (4.5)	469 549 (3.3)	2589 (6.0)	471 039 (3.3)	1099 (6.7)
No	8 375 334 (58.3)	8 246 303 (58.5)	129 031 (48.3)	8 351 615 (58.3)	23 719 (55.0)	8 365 110 (58.3)	10 224 (62.6)
Nulliparous	5 520 909 (38.41)	5 394 926 (38.3)	125 983 (47.1)	5 504 102 (38.4)	16 807 (39.0)	5 515 918 (38.4)	4991 (30.6)
Missing	4642 (0.03)	4477 (0.03)	165 (0.1)	4610 (0.03)	32 (0.1)	4635 (0.03)	7 (0.04)
Prepregnancy BMI^c^							
Underweight	462 922 (3.2)	12 624 (4.7)	12 624 (4.7)	460 943 (3.2)	1979 (4.6)	462 391 (3.2)	531 (3.3)
Normal weight	6 005 835 (41.8)	107 287 (40.1)	107 287 (40.1)	5 989 077 (41.8)	16 758 (38.8)	6 000 161 (41.79)	5674 (34.77)
Overweight	3 713 553 (25.8)	66 669 (25.0)	66 669 (25.0)	3 703 165 (25.8)	10 388 (24.1)	3 709 495 (25.84)	4058 (24.86)
Obesity	3 847 282 (26.8)	74 298 (27.8)	74 298 (27.8)	3 834 391 (26.8)	12 891 (29.9)	3 841 794 (26.8)	5488 (33.6)
Missing	343 431 (2.4)	6382 (2.4)	6382 (2.4)	342 300 (2.4)	1131 (2.6)	342 861 (2.4)	570 (3.5)
Prepregnancy diabetes							
Yes	131 262 (0.9)	129 023 (0.9)	2239 (0.8)	130 832 (0.9)	430 (1.0)	130 976 (0.9)	286 (1.8)
No	14 237 119 (99.1)	13 972 263 (99.1)	264 856 (99.1)	14 194 434 (99.1)	42 685 (98.9)	14 221 091 (99.1)	16 028 (98.2)
Missing	4642 (0.03)	4477 (0.03)	165 (0.1)	4610 (0.03)	32 (0.1)	4635 (0.03)	7 (0.04)
Prepregnancy hypertension							
Yes	277 183 (1.9)	271 666 (1.9)	5517 (2.1)	275 912 (1.9)	1271 (3.0)	276 470 (1.9)	713 (4.4)
No	14 091 198 (98.0)	13 829 620 (98.0)	261 578 (97.9)	14 049 354 (98.0)	41 844 (97.0)	14 075 597 (98.0)	15 601 (95.6)
Missing	4642 (0.03)	4477 (0.03)	165 (0.1)	4610 (0.03)	32 (0.1)	4635 (0.03)	7 (0.04)
Insurance type							
Medicaid	6 079 308 (42.3)	5 881 502 (41.7)	197 806 (74.0)	6 044 581 (42.2)	34 727 (80.5)	6 067 015 (42.3)	12 293 (75.3)
Private insurance	7 037 823 (49.0)	6 988 739 (49.6)	49 084 (18.4)	7 031 919 (49.1)	5904 (13.7)	7 035 210 (49.0)	2613 (16.0)
Self-pay	624 603 (4.3)	615 200 (4.4)	9403 (3.5)	623 555 (4.4)	1048 (2.4)	623 919 (4.3)	684 (4.2)
Other	546 214 (3.8)	536 902 (3.8)	9312 (3.5)	544 978 (3.8)	1236 (2.9)	545 593 (3.8)	621 (3.8)
Missing	85 075 (0.6)	83 420 (0.6)	1655 (0.6)	84 843 (0.6)	232 (0.5)	84 965 (0.6)	110 (0.7)
Smoking during pregnancy							
Yes	947 358 (6.6)	910 087 (6.5)	37 271 (14.0)	938 204 (6.6)	9154 (21.2)	944 797 (6.6)	2561 (15.7)
No	13 341 715 (92.8)	13 114 158 (93.0)	227 557 (85.1)	1 330 8257 (92.9)	33 458 (77.5)	13 328 225 (92.8)	13 490 (82.7)
Missing	83 950 (0.6)	81 518 (0.6)	2432 (0.9)	83 415 (0.6)	535 (1.2)	83 680 (0.6)	270 (1.7)
Initiation of prenatal care, month							
First to third	236 376 (1.6)	230 853 (1.6)	5523 (2.1)	234 878 (1.6)	1498 (3.5)	235 581 (1.6)	795 (4.9)
Fourth to sixth	10 834 011 (75.4)	10 670 148 (75.6)	163 863 (61.3)	10 808 959 (75.4)	25 052 (58.1)	10 824 614 (75.4)	9397 (57.6)
Seventh to final	2 309 105 (16.1)	2 239 640 (15.9)	69 465 (26.0)	2 297 511 (16.0)	11 594 (26.9)	2 304 995 (16.1)	4110 (25.2)
No prenatal care	648 083 (4.5)	626 017 (4.4)	22 066 (8.3)	644 410 (4.5)	3673 (8.5)	646 622 (4.5)	1461 (9.0)
Missing	345 448 (2.4)	339 105 (2.4)	6343 (2.4)	344 118 (2.4)	1330 (3.1)	344 890 (2.4)	558 (3.4)
Gestational diabetes							
Yes	914 569 (6.4)	901 968 (6.4)	12 601 (4.7)	912 698 (6.4)	1871 (4.3)	913 477 (6.4)	1092 (6.7)
No	13 453 812 (93.6)	13 199 318 (93.6)	254 494 (95.2)	13 412 568 (93.6)	41 244 (95.6)	13 438 590 (93.6)	15 222 (93.3)
Missing	4642 (0.03)	4477 (0.03)	165 (0.1)	4610 (0.03)	32 (0.1)	4635 (0.03)	7 (0.04)
Gestational hypertension or preeclampsia							
Yes	951 590 (6.6)	929 288 (6.6)	22 302 (8.3)	947 969 (6.6)	3621 (8.4)	950 056 (6.6)	1534 (9.4)
No	13 416 791 (93.5)	13 171 998 (93.4)	244 793 (91.6)	13 377 297 (93.4)	39 494 (91.5)	13 402 011 (93.4)	14 780 (90.6)
Missing	4642 (0.03)	4477 (0.03)	165 (0.1)	4610 (0.03)	32 (0.1)	4635 (0.03)	7 (0.04)
Eclampsia							
Yes	35 720 (0.3)	34 940 (0.3)	780 (0.3)	35 594 (0.3)	126 (0.3)	35 661 (0.3)	59 (0.4)
No	14 332 661 (99.7)	14 066 346 (99.7)	266 315 (99.6)	14 289 672 (99.7)	42 989 (99.6)	14 316 406 (99.7)	16 255 (99.6)
Missing	4642 (0.03)	4477 (0.03)	165 (0.1)	4610 (0.03)	32 (0.1)	4635 (0.03)	7 (0.04)
Infant sex							
Female	7 020 129 (48.8)	6 888 845 (48.8)	131 284 (49.1)	6 998 988 (48.8)	21 141 (49.0)^d^	7 012 128 (48.8)	8001 (49.0)^d^
Male	7 352 894 (51.2)	7 216 918 (51.2)	135 976 (50.9)	7 330 888 (51.2)	22 006 (51.0)^d^	7 344 574 (51.2)	8320 (51.0)^d^

Abbreviation: BMI, body mass index (calculated as weight in kilograms divided by height in meters squared).

^a^ Reported percentages are composition ratios of each horizontal item.

^b^ Other included non-Hispanic American Indian or Alaskan Native, non-Hispanic Native Hawaiian or Other Pacific Islander, non-Hispanic more than 1 race, and origin unknown or not stated.

^c^ BMI categorized as follows: underweight, <18.5; normal, 18.5-24.9; overweight, 25.0-29.9; and obesity, ≥30.

^d^ *P* > .0167 (α = .05/3), indicating that the difference did not reach statistical significance.

In the overall population, mothers who were infected with chlamydia, gonorrhea, or syphilis had an increased risk of preterm birth compared with mothers without those infections. The adjusted OR of preterm birth was 1.03 (95% CI, 1.02-1.04) for chlamydia, 1.11 (95% CI, 1.08-1.15) for gonorrhea, 1.17 (95% CI, 1.11-1.22) for syphilis, and 1.06 (95% CI, 1.05-1.07) for any STI after adjustment for age, race and ethnicity, education, marital status, parity, previous history of preterm birth, prepregnancy body mass index, prepregnancy diabetes, prepregnancy hypertension, smoking during pregnancy, initiation of prenatal care, insurance type, other infections during pregnancy, and infant sex. We further subdivided preterm birth into moderately, very, and extremely preterm birth. We found a significant association of all 3 STIs examined with moderately preterm birth. The adjusted OR of moderately preterm birth was 1.04 (95% CI, 1.02-1.05) for chlamydia, 1.10 (95% CI, 1.06-1.14) for gonorrhea, and 1.17 (95% CI, 1.11-1.23) for syphilis. Significant associations of gonorrhea and syphilis with very preterm birth were also found. The adjusted OR of very preterm birth was 1.27 (95% CI, 1.16-1.38) for gonorrhea and 1.35 (95% CI, 1.19-1.53) for syphilis (Table 2).

**Table 2.** Association Between Maternal Sexually Transmitted Infections and Preterm Birth in the US National Vital Statistics System, 2016 to 2019

Models	OR (95% CI)			
	Preterm birth	Preterm birth categories		
		Moderately	Very	Extremely
**Chlamydia**				
Model 1^a^	1.15 (1.13-1.16)	1.15 (1.14-1.17)	1.13 (1.08-1.18)	1.09 (1.04-1.15)
Model 2^b^	1.03 (1.02-1.04)	1.04 (1.02-1.05)	1.00 (0.96-1.05)	0.99 (0.93-1.04)
**Gonorrhea**				
Model 1^a^	1.36 (1.32-1.40)	1.34 (1.30-1.38)	1.53 (1.41-1.67)	1.28 (1.15-1.43)
Model 2^b^	1.11 (1.08-1.15)	1.10 (1.06-1.14)	1.27 (1.16-1.38)	1.07 (0.95-1.21)
**Syphilis**				
Model 1^a^	1.51 (1.44-1.58)	1.49 (1.41-1.56)	1.81 (1.59-2.05)	1.38 (1.15-1.65)
Model 2^b^	1.17 (1.11-1.22)	1.17 (1.11-1.23)	1.35 (1.19-1.53)	0.89 (0.72-1.08)
**Any STI**				
Model 1^a^	1.19 (1.17-1.20)	1.19 (1.17-1.20)	1.21 (1.17-1.26)	1.16 (1.10-1.21)
Model 2^b^	1.06 (1.05-1.07)	1.06 (1.05-1.08)	1.07 (1.03-1.12)	1.00 (0.95-1.05)

Abbreviations: OR, odds ratio; STI, sexually transmitted infections.

^a^ Adjusted for age, race, and ethnicity.

^b^ Model 1 plus adjustments for education, marital status, parity, previous history of preterm birth, prepregnancy body mass index, prepregnancy diabetes, prepregnancy hypertension, smoking during pregnancy, initiation of prenatal care, insurance type, other STIs during pregnancy (not applicable to the exposure “any STI”), and infant sex.

In the stratified analyses by age, race, and ethnicity, significant associations with preterm birth were observed for STI in most age groups. The adjusted OR for any STI was 1.06 (95% CI, 1.04-1.07), 1.08 (95% CI, 1.06-1.10), and 1.09 (95% CI, 1.04-1.14) among mothers younger than age 25, between ages 25 and 34, and 35 years or older, respectively. We also found STIs increased the risk of preterm birth among Hispanic (OR, 1.06; 95% CI, 1.03-1.09), non-Hispanic Black (OR, 1.04; 95% CI, 1.02-1.06), and non-Hispanic White (OR, 1.10; 95% CI, 1.08-1.13) mothers. The results for non-Hispanic Asian mothers were less stable, possibly because of the small sample size (Table 3 and Table 4).

**Table 3.** Associations Between Maternal Sexually Transmitted Infections and Preterm Birth According to Age

Age, y	Case/Total No.^a^	Adjusted OR (95% CI)^b^	*P* value for interaction
**Chlamydia**			
<25	15 554/166 216	1.04 (1.02-1.06)	.32
25-34	9409/90 231	1.03 (1.01-1.05)	
≥35	1430/10 813	1.01 (0.95-1.07)	
**Gonorrhea**			
<25	2672/24 788	1.06 (1.02-1.11)	.001
25-34	2180/16 200	1.15 (1.10-1.21)	
≥35	405/2159	1.25 (1.11-1.41)	
**Syphilis**			
<25	609/5474	1.08 (0.99-1.18)	.006
25-34	1180/8448	1.24 (1.16-1.32)	
≥35	380/2399	1.08 (0.96-1.21)	
**Any STI**			
<25	17 197/181 230	1.06 (1.04-1.07)	<.001
25-34	11 579/106 249	1.08 (1.06-1.10)	
≥35	2017/14 263	1.09 (1.04-1.14)	

Abbreviations: OR, odds ratio; STI, sexually transmitted infections.

^a^ Case totals are preterm birth cases among mothers with STIs, and the total refers to total number of mothers with STIs in the cohort.

^b^ Adjusted for race and ethnicity, education, marital status, parity, previous history of preterm birth, prepregnancy body mass index (calculated as weight in kilograms divided by height in meters squared), prepregnancy diabetes, prepregnancy hypertension, smoking during pregnancy, initiation of prenatal care, insurance type, other STIs during pregnancy (not applicable to the exposure “any STI”), and infant sex.

**Table 4.** Associations Between Maternal Sexually Transmitted Infections and Preterm Birth According to Race and Ethnicity

Race and ethnicity	Case/Total No.^a^	Adjusted OR (95% CI)^b^	*P* value for interaction
**Chlamydia**			
Hispanic	6088/72 916	1.03 (1.00-1.05)	<.001
Non-Hispanic			
Asian	292/3942	0.96 (0.84-1.08)	
Black	10 415/87 893	1.02 (1.00-1.04)	
White	7851/84 546	1.06 (1.04-1.09)	
**Gonorrhea**			
Hispanic	704/6970	1.09 (1.00-1.18)	<.001
Non-Hispanic			
Asian	37/322	1.43 (1.00-2.06)	
Black	2692/20 492	1.08 (1.03-1.13)	
White	1445/12 028	1.19 (1.12-1.26)	
**Syphilis**			
Hispanic	520/4337	1.24 (1.13-1.37)	.07
Non-Hispanic			
Asian	31/383	0.99 (0.68-1.44)	
Black	1018/6832	1.13 (1.05-1.21)	
White	462/3737	1.20 (1.08-1.33)	
**Any STI**			
Hispanic	6897/79 914	1.06 (1.03-1.09)	<.001
Non-Hispanic			
Asian	342/4459	1.01 (0.90-1.13)	
Black	12 536/103 134	1.04 (1.02-1.06)	
White	8980/93 886	1.10 (1.08-1.13)	

Abbreviations: OR, odds ratio; STI, sexually transmitted infections.

^a^ Case totals are preterm birth cases among mothers with STIs, and the total refers to total number of mothers with STIs in the cohort.

^b^ Adjusted for age, education, marital status, parity, previous history of preterm birth, prepregnancy body mass index (calculated as weight in kilograms divided by height in meters squared), prepregnancy diabetes, prepregnancy hypertension, smoking during pregnancy, initiation of prenatal care, insurance type, other STIs during pregnancy (not applicate to the exposure “any STI”), and infant sex were adjusted.

The results were robust in the sensitivity analyses that excluded pregnant women who did not receive prenatal care (eTable 2 in the Supplement) or did not have insurance or medical assistance (eTable 3 in the Supplement). In addition, the associations were consistent in sensitivity analyses using 1-year data from 2016 to 2019 (eTable 4 in the Supplement). The sensitivity analyses by additional adjustment for pregnancy complications also yielded similar results (eTable 5 in the Supplement).

## Discussion

With data from the nationwide population (over 14 million women), we found significant associations between chlamydia, gonorrhea, and syphilis and preterm birth, especially moderately or very preterm birth. The associations were relatively consistent across age and race and ethnicity groups, and the results were robust in a series of sensitivity analyses.

Previous studies about maternal STIs and preterm birth are sparse, and their findings have been inconsistent. Because of the large sample size in this population-based study, we were able to quantify the association with sufficient statistical power. Consistent with our findings, some, although not all, previous studies showed a significant association of chlamydia,^10,17,18,19^ gonorrhea,^9,10,17,18^ and syphilis^9,10,17,20^ with preterm birth. A study from California for chlamydia^9^ and another study from Washington for gonorrhea^21^ did not find any significant association with preterm birth, which may be due to the limited sample size in those studies.

The potential mechanisms between maternal STIs and preterm birth remain to be elucidated. A potential shared mechanism is inflammation. Chlamydia and gonorrhea, like some other lower genital infections, may ascend via the vagina-cervix and contribute to the occurrence of chorioamnionitis by partial activation of the systemic cytokine network.^21,22^ Syphilis, which is spread by hematogenous dissemination, may cause systemic infection and placental inflammatory response.^3,22^ All of these responses may cause inflammation and activate the maternal and/or fetal immune system, which is an established cause of preterm birth.^3,23^ However, whether a maternal infection induces preterm birth may also depend on the characteristic and concentration of the pathogen and the timing of infection.^3,5,24^

This study has important clinical and public health implications. Preterm birth is not only highly prevalent, affecting about one tenth of all births, but also clinically relevant as the leading cause of infant mortality. Therefore, it is imperative to identify modifiable and preventable risk factors for preterm birth. In recent years, there has been an increasing incidence of maternal STIs.^7^ The present study identified maternal STIs (ie, chlamydia, gonorrhea, and syphilis) as a novel risk factor for preterm birth, suggesting that addressing maternal STIs might be an unrecognized, preventive approach to reduce preterm birth. The findings support the recommendation by the CDC to screen and treat chlamydia, gonorrhea, and syphilis among pregnant women.

### Strengths and Limitations

The strengths of the current study include the use of the extremely large nationwide data set, which enabled us to estimate the risk of preterm birth in subpopulations and explore the effects of these relatively low incidence diseases on preterm birth. To the best of our knowledge, this is the first study regarding 3 STIs in relation to preterm birth in a large and diverse US population.

There are several limitations in our study. First, NVSS does not provide detailed information about treatment for these STIs. Therefore, we cannot further examine the effect modification by treatment on the association between maternal STIs and preterm birth. Second, NVSS did not specify subtypes (ie, spontaneous, medically induced, or preterm premature rupture of membranes) for each case of preterm birth. Future studies are needed to assess the association between maternal STIs and preterm birth in these clinical subtypes. Third, although we have adjusted for many factors, we cannot rule out the possibility of potential residual confounding caused by unmeasured or unknown factors. Fourth, there might be misclassification bias for maternal STIs, especially chlamydia and gonorrhea infections, because universal screening in pregnancy for these STIs is recommended but not required. Also, there was no information available to validate the diagnosis and reporting of STIs, which may introduce information bias. While the CDC and American College of Obstetricians and Gynecologists have published recommendations for screening of pregnant women for STIs, it is possible that not all clinics in the US have followed those recommendations stringently. Fifth, we don’t know the exact timing of onset for these STIs. However, we adjusted the initiation of prenatal care in our model because serologic tests for these STIs should be performed for all pregnant women and high-risk women at their first prenatal visit according to the Sexually Transmitted Diseases Treatment Guidelines in the US.

## Conclusions

In this large national study in the US, maternal STIs of chlamydia, gonorrhea, and syphilis were associated with an increased risk of preterm birth, especially of moderately and very preterm birth. These findings suggest that pregnant women with STIs before or during pregnancy warrant targeted prevention for preterm birth. Future investigation is needed to understand the underlying mechanisms.

## Notes

Supplement. **eTable 1.** Characteristics of the Study Population, According to Preterm Birth and Preterm Birth Categories **eTable 2.** Association Between Maternal Sexually Transmitted Infections and Preterm Birth, a Sensitivity Analysis by Excluding Pregnant Women Who Did Not Receive Prenatal Care **eTable 3.** Association Between Maternal Sexually Transmitted Infections and Preterm Birth, a Sensitivity Analysis by Excluding Pregnant Women Without Insurance or Medical Assistance **eTable 4.** Association Between Maternal Sexually Transmitted Infections and Preterm Birth, a Sensitivity Analysis by Including Pregnant Women in Each Year From the US National Vital Statistics System 2016-2019 **eTable 5.** Association Between Maternal Sexually Transmitted Infections and Preterm Birth, a Sensitivity Analysis by Continuing to Adjust Pregnancy Complications
