# Supplementary material for: Association of Maternal Sexually Transmitted Infections With Risk of Preterm Birth in the United States
Source: JAMA Netw Open. 2021 Nov 29;4(11):e2133413. doi: 10.1001/jamanetworkopen.2021.33413 (PMC8630565; doi:10.1001/jamanetworkopen.2021.33413)
Supplement: Supplement. — eTable 1. Characteristics of the Study Population, According to Preterm Birth and Preterm Birth Categories eTable 2. Association Between Maternal Sexually Transmitted Infections and Preterm Birth, a Sensitivity Analysis by Excluding Pregnant Women Who Did Not Receive Prenatal Care eTable 3. Association Between Maternal Sexually Transmitted Infections and Preterm Birth, a Sensitivity Analysis by Excluding Pregnant Women Without Insurance or Medical Assistance eTable 4. Association Between Maternal Sexually Transmitted Infections and Preterm Birth, a Sensitivity Analysis by Including Pregnant Women in Each Year From the US National Vital Statistics System 2016-2019 eTable 5. Association Between Maternal Sexually Transmitted Infections and Preterm Birth, a Sensitivity Analysis by Continuing to Adjust Pregnancy Complications [file jamanetwopen-e2133413-s001.pdf]

Supplemental Online Content

Gao R, Liu B, Yang W, et al. Association of maternal sexually transmitted infections with risk of preterm birth in the United States. *JAMA Netw Open.* 2021;4(11):e2133413. doi:10.1001/jamanetworkopen.2021.33413

eTable 1. Characteristics of the Study Population, According to Preterm Birth and Preterm Birth Categories

eTable 2. Association Between Maternal Sexually Transmitted Infections and Preterm Birth, a Sensitivity Analysis by Excluding Pregnant Women Who Did Not Receive Prenatal Care

eTable 3. Association Between Maternal Sexually Transmitted Infections and Preterm Birth, a Sensitivity Analysis by Excluding Pregnant Women Without Insurance or Medical Assistance

eTable 4. Association Between Maternal Sexually Transmitted Infections and Preterm Birth, a Sensitivity Analysis by Including Pregnant Women in Each Year From the US National Vital Statistics System 2016-2019

eTable 5. Association Between Maternal Sexually Transmitted Infections and Preterm Birth, a Sensitivity Analysis by Continuing to Adjust Pregnancy Complications

This supplemental material has been provided by the authors to give readers additional information about their work.

© 2021 Gao R et al. *JAMA Network Open.*

eTable 1. Characteristics of the study population, according to preterm birth and preterm birth categories

Variables	N	Preterm Birth	*P* value [a]	Preterm Birth Categories			*P* value [b]
				Moderately preterm birth (32-36 weeks)	Very preterm birth (28-31 weeks)	Extremely Preterm birth (<27 weeks)	
Overall	14373023	1146800		993679	97745	55376	
Age, years, n (%)			<0.001				<0.001
<25	3575607(24.88%)	293640(25.61%)		252167(25.37%)	25739(26.34%)	15734(28.41%)	
25-34	8270420(57.55%)	615836(53.70%)		535849(53.92%)	51240(52.42%)	28747(51.91%)	
≥35	2526996(17.58%)	237324(20.70%)		205663(20.70%)	20766(21.25%)	10895(19.68%)	
Race or ethnicity, n (%)			<0.001				<0.001
Hispanic	3435333(23.90%)	276833(24.14%)		241519(24.31%)	22304(22.82%)	13010(23.49%)	
Non-Hispanic White	7386568(51.39%)	519937(45.34%)		462020(46.50%)	39385(40.29%)	18532(33.47%)	
Non-Hispanic Black	2058006(14.32%)	235628(20.55%)		190103(19.13%)	26581(27.19%)	18944(34.21%)	
Non-Hispanic Asian	912425(6.35%)	63452(5.53%)		56032(5.64%)	5015(5.13%)	2405(4.34%)	
Other	580691(4.04%)	50950(4.44%)		44005(4.43%)	4460(4.56%)	2485(4.49%)	
Education levels, n (%)			<0.001				<0.001
Lower than high school	1864949(12.98%)	179161(15.62%)		154567(15.56%)	15770(16.13%)	8824(15.93%)	
High school	3655819(25.44%)	327172(28.53%)		280518(28.23%)	29082(29.75%)	17572(31.73%)	
Higher than high school	8667305(60.30%)	623597(54.38%)		544393(54.79%)	51246(52.43%)	27958(50.49%)	
Missing	184950(1.29%)	16870(1.47%)		14201(1.43%)	1647(1.68%)	1022(1.85%)	
Marital status, n (%)			<0.001				<0.001
Yes	7797727(54.25%)	545636(47.58%)		481830(48.49%)	42007(42.98%)	21799(39.37%)	
No	5256032(36.57%)	510771(44.54%)		432668(43.54%)	48309(49.42%)	29794(53.80%)	
Missing	1319264(9.18%)	90393(7.88%)		79181(7.97%)	7429(7.60%)	3783(6.83%)	
Parity, n (%)			<0.001				<0.001
1	5522842(38.43%)	443629(38.68%)		374275(37.67%)	42485(43.47%)	26869(48.52%)	

© 2021 Gao R et al. *JAMA Network Open.*

2	4582467(31.88%)	315715(27.53%)		278124(27.99%)	24478(25.04%)	13113(23.68%)	
3	2437738(16.96%)	195400(17.04%)		172712(17.38%)	15090(15.44%)	7598(13.72%)	
≥4	1793349(12.48%)	188191(16.41%)		165399(16.65%)	15265(15.62%)	7527(13.59%)	
Missing	36627(0.25%)	3865(0.34%)		3169(0.32%)	427(0.44%)	269(0.49%)	
Previous preterm birth, n (%)			<0.001				<0.001
Yes	472138(3.28%)	124251(10.83%)		105634(10.63%)	12259(12.54%)	6358(11.48%)	
No	8375334(58.27%)	578603(50.45%)		513500(51.68%)	42969(43.96%)	22134(39.97%)	
Nulliparous	5520909(38.41%)	443342(38.66%)		374043(37.64%)	42462(43.44%)	26837(48.46%)	
Missing	4642(0.03%)	604(0.05%)		502(0.05%)	55(0.06%)	47(0.08%)	
Pre-pregnancy BMI, n (%)			<0.001				<.001
Underweight	462922(3.22%)	44472(3.88%)		38912(3.92%)	3752(3.84%)	1808(3.26%)	
Normal weight	6005835(41.79%)	422542(36.85%)		371674(37.40%)	33647(34.42%)	17221(31.10%)	
Overweight	3713553(25.84%)	279449(24.37%)		243031(24.46%)	23278(23.82%)	13140(23.73%)	
Obesity	3847282(26.77%)	358599(31.27%)		306442(30.84%)	32129(32.87%)	20028(36.17%)	
Missing	343431(2.39%)	41738(3.64%)		33620(3.38%)	4939(5.05%)	3179(5.74%)	
Pre-pregnancy diabetes, n (%)			<0.001				<0.001
Yes	131262(0.91%)	33653(2.93%)		29173(2.94%)	3126(3.20%)	1354(2.45%)	
No	14237119(99.05%)	1112543(97.01%)		964004(97.01%)	94564(96.75%)	53975(97.47%)	
Missing	4642(0.03%)	604(0.05%)		502(0.05%)	55(0.06%)	47(0.08%)	
Pre-pregnancy hypertension, n (%)			<0.001				<0.001
Yes	277183(1.93%)	59317(5.17%)		48253(4.86%)	7335(7.50%)	3729(6.73%)	
No	14091198(98.04%)	1086879(94.77%)		944924(95.09%)	90355(92.44%)	51600(93.18%)	
Missing	4642(0.03%)	604(0.05%)		502(0.05%)	55(0.06%)	47(0.08%)	
Smoking during pregnancy, n (%)			<0.001				<0.001
Yes	947358(6.59%)	111653(9.74%)		96318(9.69%)	10148(10.38%)	5187(9.37%)	
No	13341715(92.82%)	1008533(87.94%)		890884(89.66%)	86738(88.74%)	30911(55.82%)	
Missing	83950(0.58%)	26614(2.32%)		6477(0.65%)	859(0.88%)	19278(34.81%)	
Initiation of prenatal care, n (%)			<0.001				<0.001

© 2021 Gao R et al. *JAMA Network Open.*

1st to 3rd month	236376(1.64%)	52985(4.62%)		41527(4.18%)	6615(6.77%)	4843(8.75%)	
4th to 6th month	10834011(75.38%)	828383(72.23%)		722327(72.69%)	67938(69.51%)	38118(68.83%)	
7th to final month	2309105(16.07%)	174247(15.19%)		151134(15.21%)	15086(15.43%)	8027(14.50%)	
No prenatal care	648083(4.51%)	41806(3.65%)		40051(4.03%)	1718(1.76%)	37(0.07%)	
Missing	345448(2.40%)	49379(4.31%)		38640(3.89%)	6388(6.54%)	4351(7.86%)	
Gestational diabetes, n (%)			<0.001				<0.001
Yes	914569(6.36%)	103649(9.04%)		94339(9.49%)	7173(7.34%)	2137(3.86%)	
No	13453812(93.60%)	1042547(90.91%)		898838(90.46%)	90517(92.61%)	53192(96.06%)	
Missing	4642(0.03%)	604(0.05%)		502(0.05%)	55(0.06%)	47(0.08%)	
Gestational hypertension or preeclampsia, n (%)			<0.001				<0.001
Yes	951590(6.62%)	184036(16.05%)		157777(15.88%)	19529(19.98%)	6730(12.15%)	
No	13416791(93.35%)	962160(83.90%)		835400(84.07%)	78161(79.96%)	48599(87.76%)	
Missing	4642(0.03%)	604(0.05%)		502(0.05%)	55(0.06%)	47(0.08%)	
Eclampsia, n (%)			<0.001				<0.001
Yes	35720(0.25%)	12941(1.13%)		10215(1.03%)	1953(2.00%)	773(1.40%)	
No	14332661(99.72%)	1133255(98.82%)		982962(98.92%)	95737(97.95%)	54556(98.52%)	
Missing	4642(0.03%)	604(0.05%)		502(0.05%)	55(0.06%)	47(0.08%)	
Insurance type, n (%)			<0.001				<0.001
Medicaid	6079308(42.30%)	562415(49.04%)		482822(48.59%)	50234(51.39%)	29359(53.02%)	
Private Insurance	7037823(48.97%)	490067(42.73%)		429783(43.25%)	39227(40.13%)	21057(38.03%)	
Self-Pay	624603(4.35%)	43592(3.80%)		37434(3.77%)	3744(3.83%)	2414(4.36%)	
Other	546214(3.80%)	43008(3.75%)		37119(3.74%)	3810(3.90%)	2079(3.75%)	
Missing	85075(0.59%)	7718(0.67%)		6521(0.66%)	730(0.75%)	467(0.84%)	
Infant sex, n (%)			<0.001				<0.001
Female	7020129(48.84%)	529869(46.20%)		458309(46.12%)	45247(46.29%)	26313(47.52%)	
Male	7352894(51.16%)	616931(53.80%)		535370(53.88%)	52498(53.71%)	29063(52.48%)	
Chlamydia Infection, n (%)			<0.001				<0.001
Yes	267260(1.86%)	26393(2.30%)		22448(2.26%)	2430(2.49%)	1515(2.74%)	

© 2021 Gao R et al. *JAMA Network Open.*

No	14105763(98.14%)	1120407(97.70%)		971231(97.74%)	95315(97.51%)	53861(97.26%)	
Gonorrhea Infection, n (%)			<0.001				<0.001
Yes	43147(0.30%)	5257(0.46%)		4346(0.44%)	585(0.60%)	326(0.59%)	
No	14329876(99.70%)	1141543(99.54%)		989333(99.56%)	97160(99.40%)	55050(99.41%)	
Syphilis Infection, n (%)			<0.001				<0.001
Yes	16321(0.11%)	2169(0.19%)		1796(0.18%)	250(0.26%)	123(0.22%)	
No	14356702(99.89%)	1144631(99.81%)		991883(99.82%)	97495(99.74%)	55253(99.78%)	

P value [a]: Difference between full-term and preterm birth.
P value [b]: Difference among full-term, moderately preterm birth, very preterm birth and extremely preterm birth.

© 2021 Gao R et al. *JAMA Network Open.*

eTable 2. Association between maternal sexually transmitted infections and preterm birth, a sensitivity analysis by excluding pregnant women who did not receive prenatal care

	Preterm birth，OR (95% CI)	
	**Model1**	**Model2**
Chlamydia	1.15 (1.13-1.17)	1.02 (1.01-1.04)
Gonorrhea	1.30 (1.26-1.35)	1.10 (1.06-1.13)
Syphilis	1.40 (1.33-1.47)	1.13 (1.08-1.19)
Any STI	1.18 (1.17-1.20)	1.05 (1.03-1.06)

Model1: Age, race/ethnicity were adjusted in this model.
Model2: model1+ education, marital status, parity, previous history of preterm birth, pre-pregnancy body mass index, pre-pregnancy diabetes, pre-pregnancy hypertension, smoking during pregnancy, insurance type, other STIs during pregnancy (not applicate to the exposure "any STIs") and infant sex were adjusted.

© 2021 Gao R et al. *JAMA Network Open.*

eTable 3. Association between maternal sexually transmitted infections and preterm birth, a sensitivity analysis by excluding pregnant women without insurance or medical assistance

	Preterm birth，OR (95% CI)	
	**Model1**	**Model2**
Chlamydia	1.14 (1.13-1.16)	1.03 (1.02-1.04)
Gonorrhea	1.34 (1.30-1.38)	1.11 (1.07-1.14)
Syphilis	1.48 (1.41-1.55)	1.16 (1.10-1.22)
Any STI	1.18 (1.17-1.19)	1.06 (1.05-1.07)

Model1: Age, race/ethnicity were adjusted in this model.
Model2: model1+ education, marital status, parity, previous history of preterm birth, pre-pregnancy body mass index, pre-pregnancy diabetes, pre-pregnancy hypertension, smoking during pregnancy, initiation of prenatal care, other STIs during pregnancy (not applicate to the exposure “any STIs”) and infant sex were adjusted.

© 2021 Gao R et al. *JAMA Network Open.*

eTable 4. Association between maternal sexually transmitted infections and preterm birth, a sensitivity analysis by including pregnant women in each year from the US National Vital Statistics System 2016-2019

	Year	Preterm birth，OR (95% CI)	
		**Model1**	**Model2**
Chlamydia	2019	1.14 (1.11-1.17)	1.01 (0.99-1.04)
Gonorrhea		1.39 (1.31-1.46)	1.12 (1.05-1.19)
Syphilis		1.73 (1.60-1.86)	1.31 (1.21-1.42)
Any STI		1.21 (1.18-1.24)	1.07 (1.04-1.09)
Chlamydia	2018	1.17 (1.14-1.20)	1.04 (1.01-1.07)
Gonorrhea		1.40 (1.32-1.48)	1.11 (1.05-1.18)
Syphilis		1.54 (1.41-1.68)	1.16 (1.06-1.27)
Any STI		1.20 (1.18-1.23)	1.07 (1.04-1.09)
Chlamydia	2017	1.15 (1.12-1.19)	1.03 (1.00-1.06)
Gonorrhea		1.35 (1.27-1.44)	1.12 (1.05-1.20)
Syphilis		1.34 (1.21-1.49)	1.06 (0.95-1.18)
Any STI		1.18 (1.16-1.21)	1.06 (1.03-1.09)
Chlamydia	2016	1.14 (1.11-1.17)	1.03 (1.00-1.06)
Gonorrhea		1.28 (1.21-1.37)	1.08 (1.01-1.15)
Syphilis		1.27 (1.14-1.41)	1.02 (0.91-1.14)
Any STI		1.15 (1.13-1.18)	1.05 (1.02-1.07)

Model1: Age, race/ethnicity were adjusted in this model.
Model2: model1+ education, marital status, parity, previous history of preterm birth, pre-pregnancy body mass index, pre-pregnancy diabetes, pre-pregnancy hypertension, smoking during pregnancy, initiation of prenatal care, other STIs during pregnancy (not applicate to the exposure “any STIs”) and infant sex were adjusted.

© 2021 Gao R et al. *JAMA Network Open.*

eTable 5. Association between maternal sexually transmitted infections and preterm birth, a sensitivity analysis by continuing to adjust pregnancy complications

	Preterm birth, OR (95% CI)	Preterm Birth Categories		
		Moderately preterm birth, OR (95% CI)	Very preterm birth, OR (95% CI)	Extremely preterm birth, OR (95% CI)
Chlamydia	1.05 (1.03-1.06)	1.02(1.00-1.03)	0.98(0.94-1.02)	0.98(0.93-1.04)
Gonorrhea	1.17 (1.14-1.21)	1.10(1.06-1.14)	1.26(1.16-1.37)	1.07(0.94-1.21)
Syphilis	1.21 (1.15-1.27)	1.14(1.08-1.20)	1.30(1.15-1.48)	0.87(0.71-1.06)
Any STI	1.09 (1.07-1.10)	1.05(1.03-1.06)	1.05(1.01-1.09)	0.99(0.94-1.05)

Model2+ pregnancy complications.
Pregnancy complications was defined as having gestational diabetes, gestational hypertension, preeclampsia, or eclampsia.

© 2021 Gao R et al. *JAMA Network Open.*
